# Transcriptome Analysis of *C. elegans* Reveals Novel Targets for DON Cytotoxicity

**DOI:** 10.3390/toxins10070262

**Published:** 2018-06-27

**Authors:** Rong Di, Hanzhong Zhang, Michael A. Lawton

**Affiliations:** Department of Plant Biology, Rutgers, The State University of New Jersey, New Brunswick, NJ 08901, USA; rongdi@sebs.rutgers.edu (R.D.); hzhang1234@aol.com (H.Z.)

**Keywords:** *Fusarium* head blight, deoxynivalenol, cytotoxicity, *Caenorhabditis elegans*, RNAseq

## Abstract

Deoxynivalenol (DON) is a mycotoxin produced by *Fusarium* spp. that causes *Fusarium* head blight (FHB) disease in cereal crops. Ingestion of food contaminated with DON poses serious human health complications. However, the DON cytotoxicity has been mostly deduced from animal studies. In this study, we used the nematode *Caenorhabditis elegans* (*C. elegans*) as a tractable animal model to dissect the toxic effect of DON. Our results indicate that DON reduces the fecundity and lifespan of *C. elegans*. Real-time quantitative polymerase chain reaction (RT-qPCR) analysis showed that DON upregulates innate immunity-related genes including *C17H12.8* and *K08D8.5* encoding PMK-1 (mitogen activated protein kinase-1)-regulated immune effectors, and *F35E12.5* encoding a CUB-like domain-containing protein. Furthermore, our RNAseq data demonstrate that out of ~17,000 *C. elegans* genes, 313 are upregulated and 166 were downregulated by DON treatment. Among the DON-upregulated genes, several are *ugt* genes encoding UDP-glucuronosyl transferase (UGTs) which are known to be involved in chemical detoxification. The three upregulated genes, *F52F10.4* (*oac-32*), *C10H11.6* (*ugt-26*) and *C10H11.4* (*ugt-28*) encoding the O-acyltransferase homolog, UGT26 and UGT 28, respectively, are shown to contribute to DON tolerance by a RNAi bacterial feeding experiment. The results of this study provide insights to the targets of DON cytotoxicity and potential mitigation measures.

## 1. Introduction

Deoxynivalenol (DON), a trichothecene mycotoxin, is produced by species of the fungus *Fusarium* which is responsible for causing *Fusarium* head blight (FHB) disease in wheat, barley, maize, oats, rye and rice worldwide [1,2]. *Fusarium* spp. infection of plants results in both yield loss and the contamination of grain with mycotoxins. It has been shown that acute exposure of experimental animals to extremely high doses (≥27 mg/kg body weight) of DON in diets causes mortality or marked tissue injury, whereas acute exposure to relatively low doses (≥50 g/kg body weight) induces vomiting in the most sensitive pigs [3]. Chronic exposure of pigs to 2 ppm of DON impairs weight gain and disrupts immune functions [3].

As reviewed by Pestka [4], the mechanisms of DON cytotoxicity mainly involve the modulation of the innate immune systems by interacting with ribosomes, activating double-stranded RNA-associated protein kinase (PKR), inhibiting translation, evoking endoplasmic reticulum (ER) stress responses and elevating the expression of proinflammatory genes. Most of the known toxic effects of DON and its cytotoxic mechanisms have been studied in experimental animals such as pigs [3] and mice [5]. Besides a limited number of epidemiological studies, DON cytotoxicity in humans has been deduced mainly from studies performed in animals [2]. Since pigs have been used as a model to study human intestines [6] and drug-induced emesis [7], it has been suggested that people may be as sensitive to DON as are pigs, with a vomiting threshold of 50–100 g/kg body weight [2]. Nonetheless, significant questions remain regarding the cytotoxic mechanisms of DON in humans and the mitigation strategies that might counter its effects.

The nematode *Caenorhabditis elegans* (*C. elegans*) is a popular model organism for genetic and developmental studies owing to its sequenced genome, well-defined cell lineage, the availability of an extensive knockout mutant library, and the high degree conservation of gene functions and signal transduction pathways with humans [8]. It has also been used as an invertebrate model for toxicological studies for heavy metals [9], new chemicals [10] and known toxicants such as paraquat and parathion [11]. *C. elegans* was first used in 2011 to show that the brood size and developmental rate of *C. elegans* were significantly affected when exposed to 500 and 1000 g/mL of DON [12]. The toxic effects of DON on the growth, reproduction and lifespan of *C. elegans* were further studied recently [13]. However, the precise mechanisms of DON cytotoxicity in *C. elegans* remain unclear.

In order to understand the overall toxic effects of DON in *C. elegans*, we employed RNAseq to analyze the comprehensive modulation of gene expression in *C. elegans* upon DON intoxication. RNAseq analysis has been used to elucidate transcriptional changes following manganese exposure in *C. elegans* [14]. Whole genome microarray analysis has similarly been used to profile the host response in mouse brain following exposure to the plant toxin abrin [15]. Our results indicate that exposure of *C. elegans* to DON led to the upregulation of genes involved in innate immunity and the downregulation of structural and housekeeping genes. These data provide the molecular foundation for developing a better understanding of the mechanisms of DON toxicity in the *C. elegans* model and for identifying possible targets for the intervention and amelioration of DON toxicity in animals and humans.

## 2. Results

### 2.1. Deoxynivalenol (DON) Reduces Fecundity and Lifespan of C. elegans

**Figure 1:**
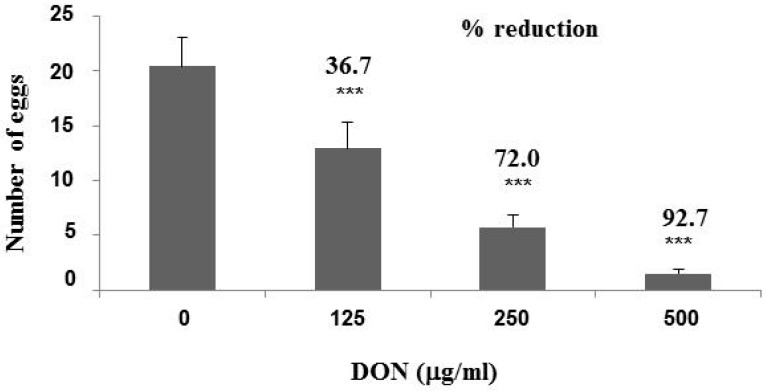
Effect of deoxynivalenol (DON) toxicity on egg-laying. N2 worms at the L2 stage were treated with the indicated concentrations of DON for 24 h. After transfer onto nematode growth medium (NGM) without DON, they were allowed to lay eggs for 24 h. Egg number was counted from at least 30 individual worms in each treatment and averaged from three independent experiments. Student’s *t*-test was used to analyze the significant difference (***, *p* < 0.001) among treatments.

**Figure 2:**
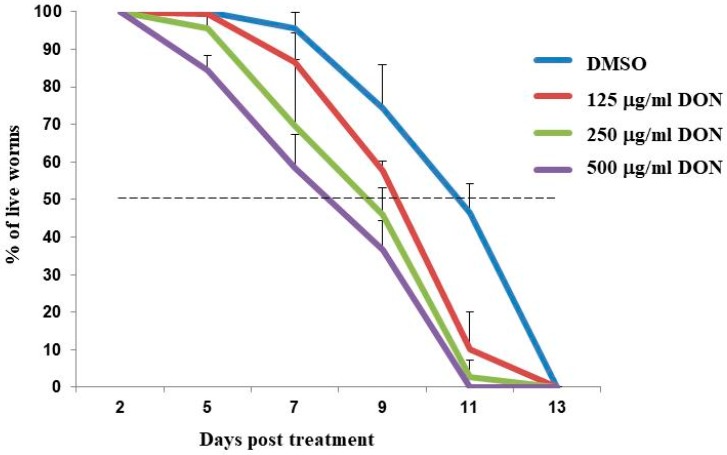
Effect of DON toxicity on lifespan. N2 worms at L2 stage were treated with 125, 250 and 500 μg/mL DON for 24 h. After placing L4 worms on NGM medium containing 0.1 mg/mL 5-fluorodeoxyuridine (FUDR), the worms’ viability was recorded every two days.

Synchronized wild type N2 worms at the L2 stage were exposed to 125, 250 and 500 μg/mL DON for 24 h. Exposure to 250 and 500 μg/mL DON reduced viability rate of N2 worms from 100% to 95–98%, while exposure to 125 μg/mL DON had no effect. Although there was only a minor effect on L2 stage worm viability, exposure to DON resulted in a marked reduction in fecundity. Transfer of L4 stage worms to nematode growth medium (NGM) showed that DON exposure significantly (*p* < 0.001) reduced the number of eggs produced 24 h later by 36.7%, 72.0% and 92.7%, respectively (Figure 1). Worms exposed to DON in 0.6% dimethyl sulfoxide (DMSO) only were placed onto NGM medium containing 0.1 mg/mL 5-fluorodeoxyuridine (FUDR) to prevent production of additional progeny, allowing the effect of DON toxicity on the worm’s lifespan to be observed (Figure 2). Treatment of worms with 500 μg/mL DON resulted in 100% mortality at 11 days post treatment (dpt), whereas worms treated with only 0.6% DMSO (control sample) took until 13 dpt to reach 100% mortality. At 11 dpt, the mortality rate for worms treated with 250 and 125 μg/mL DON were 98% and 90%, respectively. It took 11.5 days to result in 50% mortality for the 0.6% DMSO-fed worms, however, only 8.5, 9.0 and 9.5 days were needed, respectively, for the 500, 250 and 125 μg/mL DON-treated worms to reach 50% mortality (Figure 2).

### 2.2. DON Upregulates Genes Involved in Innate Immunity in *C. elegans*

We examined DON-induced changes in mRNA levels of specific transcripts that were previously shown to be induced following exposure to bacterial pathogens [16]. Expression of the genes *C17H12.8* and *K08D8.5*, encoding PMK-1 (mitogen activated protein kinase-1)-regulated immune effectors, are upregulated after is exposed to *Pseudomonas aerugunosa* strain PA14 [16] and to Shiga toxin-producing enterohaemorrhagic *Escherichia coli* (*E. coli*) strain EDL933 [17]. We initially studied whether DON also induces expression of these two genes, as well two other genes involved in innate immunity (*R03G5.2*, which encodes *sek-1* and functions in activation of MAPK activity [17]; and *F35E12.5*, which encodes a CUB-like domain-containing protein) [18]. Treatment with the alkaloid compound harmane has been shown to upregulate *F35E12* expression by approximately 8-fold during *E. coli* EDL933 infection and extend lifespan of [18].

**Figure 3:**
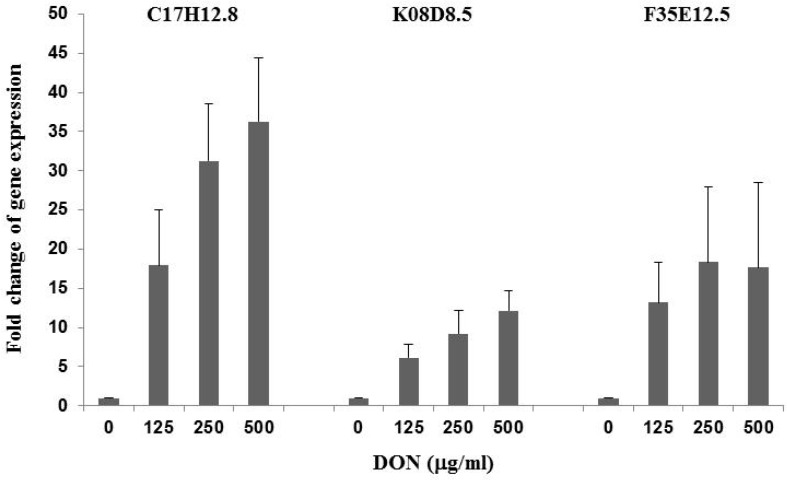
Upregulation of innate immunity-related genes in by DON. Real-time RT-qPCR analysis was conducted after worms were treated for 24 h with different levels of DON. Gene expression levels were compared to worms treated with 0.6% dimethyl sulfoxide (DMSO) by the 2^−ΔΔCt^ relative quantification method. Actin 1 (*T04C12.6.2*) was used as the endogenous control gene.

*C. elegans* worms at L2 stage were treated with 125, 250 and 500 μg/mL DON for 24 h. Real-time quantitative polymerase chain reaction (RT-qPCR) analysis using total worm RNA as template and gene-specific primers (Table 1) showed that *R03G5.2* gene expression was not affected by DON treatment. However, the mRNA levels of *C17H12.8*, *K08D8.5* and *F35E12.5* were substantially upregulated by DON. Figure 3 shows that expression of the *C17H12.8* gene increased in a dose-dependent manner by 16.9-, 30.3- and 35.3-fold when *C. elegans* was exposed to 125, 250 and 500 μg/mL DON, respectively, compared to comparable treatment with 0.6% DMSO. *F35E12.5* gene expression increased by 12.2-, 17.4- and 16.7-fold when worms were, respectively, exposed to 125, 250 and 500 μg/mL DON. Gene expression of *K0.8Δ8.5* was also induced but less substantially, compared to *C17H12.8* and *F35E12.5*, with 5.2-, 8.2- and 11.1-fold increases following the exposure of worms to 125, 250 and 500 μg/mL DON, respectively.

### 2.3. Gene Global Expression is Modulated by DON in RNAseq Analysis

**Figure 4:**
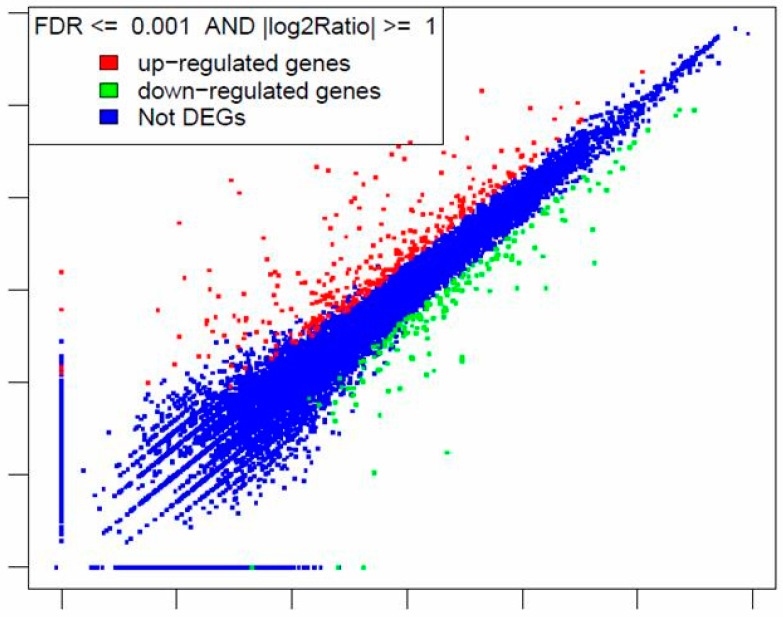
Scatter plot of differentially expressed genes in after treatment with 250 μg/mL DON for 24 h. Gene expression was analyzed by RNAseq.

To investigate whether DON affects the expression of additional genes involved in innate immunity as well as genes associated with other cellular functions, we treated the L2 worms with either 250 μg/mL DON or 0.6% DMSO (as control) for 24 h, extracted total RNA from each sample and characterized by RNAseq (performed by BGI). Following RNAseq analysis and gene annotation, gene expression levels from technical replicate samples were analyzed and averaged. As shown by the scatter plot of differentially expressed genes (DEGs) (Figure 4), out of ~17,000 genes, 313 genes were upregulated and 166 genes were downregulated by DON treatment. Table 2 lists 43 genes upregulated by more than 10-fold and the ten genes downregulated by more than 10-fold. The complete RNAseq data for all genes of *C. elegans* are included in Table S1.

Table 2 shows that the expression of *C17H12.8, K08D8.5* and *F35E12.8*, three genes involved in innate immunity, increased, respectively, 32.4-, 12.0- and 11.8-fold as shown by RNAseq analysis and 30.3-, 8.2- and 17.4-fold as shown by RT-qPCR assay performed on a separate batch of treated worms (Figure 3). Additionally, both RNAseq and RT-qPCR analysis showed that the *R03G5.2* (*sek-1*) gene expression was not affected by 250 μg/mL DON treatment. These comparative data provide support for the validity of the RNAseq results for genes whose expression has not been previously validated by RT-qPCR.

The *C. elegans* database WormBase (WormBase.org) shows that the genome contains 66 *ugt* genes encoding UDP-glucuronosyl transferase (UGT). UGTs are involved in chemical detoxification and are present in organisms of all the major kingdoms [19]. The RNAseq results indicate that the *C. elegans ugt-31*, *ugt-26*, *ugt-29* and *ugt-28* gene expression was upregulated by DON by 39.5-, 37.1-, 33.1- and 27.8-fold respectively (Table 2). Additionally, the RNAseq results showed that the expression of *ugt-16*, *ugt-24*, *ugt-48*, *ugt-19*, *ugt-62* and *ugt-1* genes were increased by 6.2-, 5.5-, 4.8-, 4.4-, 4.1- and 2.6-fold, respectively, upon DON treatment.

Our RNAseq data also indicate that 10 genes were downregulated at least 10-fold upon DON treatment, including four genes encoding collagen (Table 2).

### 2.4. Functional Analysis of DON-Induced Genes in Conferring DON Tolerance

We used an RNAi suppression assay to assess the functional contributions of DON-induced genes to the ability of worms to cope with exposure to DON. We chose three genes belonging to the group whose expression was most highly upregulated by DON intoxication (Table 2). RNAi-expressing bacteria, obtained from Open Biosystems, were fed to worms and used to suppress the expression of: *F52F10.4* (*oac-32*) encoding the *O*-acyltransferase homolog, *C10H11.6* (*ugt-26*) encoding UDP-glucuronosyl transferase 26 and *C10H11.4* (*ugt-28*) encoding UDP-glucuronosyl transferase 28. Synchronized eggs were placed on NGM plates seeded with either OP50 bacteria or *oac-32*, *ugt-26* and *ugt-28* RNAi bacteria, and the worms were grown for 24 h. The L2 worms (around 50 for each treatment) were then treated with 250 μg/mL DON in S-medium supplemented with OP50 or *oac-32*, *ugt-26* or *ugt-28* RNAi bacteria for 24 h. This experiment was repeated three times, standard errors were expressed as “±”. As a control, L2 worms were cultured in S-medium lacking DON and exposed to either OP50, *oac-32*, *ugt-26* or *ugt-28* RNAi bacteria for 24 h. There was no viability reduction in the worms cultured with *oac-32*, *ugt-26* or *ugt-28* RNAi bacteria, compared to those cultured in OP50 (data not shown), indicating that suppression of these genes has no effect on the normal lifespan of worms. Counting the number of live worms (at L4 stage now) at the end of DON treatment showed that worm viability was reduced to 89.3% ± 8.1% (*p* = 0.04), 84.3% ± 4.0% (*p* = 0.001) and 91.7% ± 2.9% (*p* = 0.003), respectively, when fed with *oac-32*, *ugt-26* or *ugt-28* RNAi cells, compared to a viability of 97.7% ± 2.5% (*p* = 0.09) when fed with OP50 and a viability of 100% when not treated with DON at all. These data demonstrate that the suppression of *oac-32*, *ugt-26* and *ugt-28* genes reduced *C. elegans*’ capability to resist DON toxicity, indicating the possible contribution of these genes to the detoxification mechanisms of *C. elegans*.

**Figure 5:**
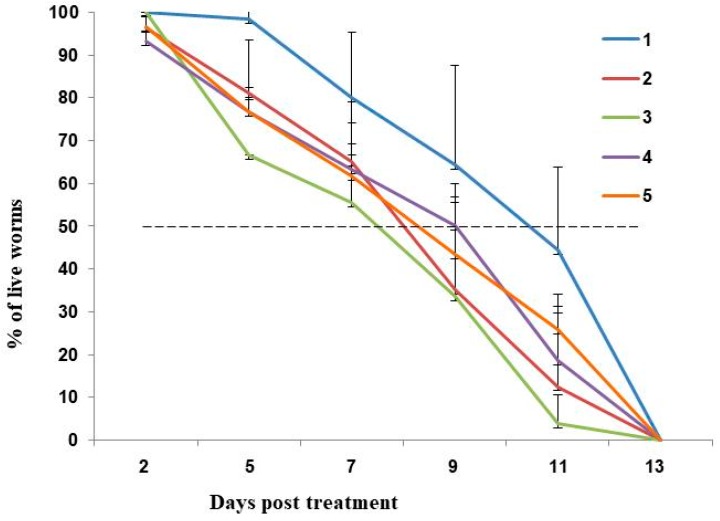
Lifespan analysis of worms initially fed with OP50 or *oac-32*, *ugt-26* and *ugt-28* RNAi bacteria and then treated with 250 μg/mL DON. After placing L4 worms on NGM medium containing 0.1 mg/mL FUDR and OP50, the worm’s viability was recorded every two days. 1, OP50; 2, DON + OP50; 3, DON + *oac-32* RNAi; 4, DON + *ugt-26* RNAi; 5, DON + *ugt-28* RNAi. The results were averaged from three independent biological experiments.

We then monitored the lifespan of the L4 worms that survived the DON treatment on NGM containing FUDR and seeded with OP50 bacteria. Worms fed with OP50 reached 50% mortality 9.3 days after initial exposure to DON, compared to 10.5 days in the absence of DON (X-axis, Figure 5). Worms fed with *oac-32*, *ugt-26* and *ugt-28* RNAi bacteria reached 50% mortality rates at 7.4, 7.2 and 7.5 days, respectively. These data indicate that suppression of the *oac-32*, *ugt-26* and *ugt-28* genes (potentially involved in innate immunity and detoxicification) slightly decreases the lifespan of and that these genes may contribute to the ability of *C. elegans* to respond to and cope with the effects of DON toxicity. However, the small effects observed through the suppression of these genes indicates that there may be other genes whose contribution to the response to DON is more significant, or that there is redundancy in the transcriptional response to DON.

## 3. Discussion

In order to better understand the molecular mechanisms of DON cytotoxicity, a trichothecene toxin present on grain crops infected with *Fusarium* spp., we examine global changes in DON-induced transcription in the model organism *C. elegans*. It has been shown by Gowrinathan et al. [12] that N2 display lower rates of development and smaller brood sizes when exposed to 500 and 100 μg/mL DON. In this study, we showed systematically that the N2 worms’ egg-laying capability was significantly reduced by 125, 250 and 500 μg/mL DON in a dose-dependent manner (Figure 1). We also showed that worm lifespan was reduced following exposure to 250 μg/mL DON, with worms requiring only 8.51 days to reach 50% mortality, compared to 11.45 days for control worms (Figure 2). Yang et al. [13] recently showed that 4 days were needed to approach 50% mortality of N2 worms exposed to 65.67 μg/mL DON, compared to the 16 days needed for control worms. However, because the developmental stage and the culture conditions used were not indicated in that report, it is not possible to compare our findings directly with this previous study.

It has been suggested that innate immunity may play a role in the molecular response of animals and humans to DON cytotoxicity [4]. It has been shown, previously, that the expression of immune effector genes *C17H12.8*, *K08D8.5* [17] and *F35E12.5* are upregulated following exposure to infection by the Shiga toxin-producing *E. coli* O157:H7 strain EDL933 [18]. We investigated changes in the expression of these three genes in *C. elegans* exposed to DON to see if they are similarly affected by DON. Our data show that all three genes are highly upregulated in a DON-dosage dependent manner (Figure 3), indicating that components of the worm’s innate immunity are modulated by DON. We used RNAseq to investigate changes in the pattern of global gene expression in exposed to 250 μg/mL DON.

To our knowledge, this is the first time the toxic effects of DON on *C. elegans* gene expression have been comprehensively investigated. Our data showed that out of ~17,000 *C. elegans* genes, 313 genes were upregulated and 166 genes were downregulated by DON treatment (Figure 4). The *F11D11.3* gene was upregulated by 612.6-fold; interestingly, this gene is listed with no known molecular function in the WormBase (WormBase.org), suggesting that the approach adopted here of global transcriptome analysis may help identify novel components of the response to DON toxicity.

Another interesting result from our RNAseq analysis is that out of 66 *ugt* genes found in the WormBase, 10 *ugt* genes were upregulated at least 2-fold. Of these, expression of *ugt-31*, *ugt-26*, *ugt-29* and *ugt-28* increased by more than 20-fold (Table 2). The *ugt* genes encode uridine diphosphate (UDP)-glycosyltransferases and the encoded enzymes carry out hexosyl group transfer, a common mechanism for detoxification of xenobiotics. A superfamily of more than 100 *ugt* genes has been identified in the model plant *Arabidopsis thaliana* [20]. However, very little is known about the regulation and localization of these plant UGTs [20]. Poppenberger et al. have shown that the enzymatic reaction of Arabidopsis *UGT73C5* (locus At2g36800) could inactivate DON in yeast and wheat germ, and that constitutive overexpression of this gene in Arabidopsis led to enhanced resistance against DON [21]. A later report showed that transgenic Arabidopsis plants expressing a barley *ugt* gene (*HvUGT13248*) were more resistant to DON in a seed germination assay and converted DON to DON-3-*O*-glucoside (D3G) to a greater extent, compared to the wild-type plants [22]. Additionally, transgenic wheat plants expressing *HvUGT13248* have been shown to detoxify DON to D3G, better tolerate DON in roots and exhibit less severe disease symptoms in the field, compared to non-transformed wheat plants [23]. Together, these reports suggest that the up-regulation of *ugt* genes may be similarly important in DON detoxification in as they are in plants. However, the size of the *ugt* gene family in *C. elegans* and the fact that 10 members were induced by DON suggests that there may be functional redundancy.

This study has provided a comprehensive picture of the gene perturbation by DON and shed light on potential targets in plants for FHB disease resistance and in animals for developing therapeutic measures. The specificity of the response (comprising both induced and suppressed genes) indicates that it may be possible to ameliorate DON toxicity through manipulation of relatively few molecular targets. Further functional analysis of these targets, through RNAi suppression and through gene disruption experiments may prove informative, in this regard.

## 4. Materials and Methods

### 4.1. Materials and Maintenance

DON was purchased from Sigma Aldrich (St. Louis, MO, USA) and diluted in a final concentration of 0.6% DMSO for all samples tested. DMSO below 1% is generally used and has been regarded as not affecting nematode growth and development [13]. Wild-type N2 *C. elegans* worms were obtained from the *Caenorhabditis* Genetics Center (CGC, St. Paul, MN, USA). Worms were maintained on NGM with the OP50 strain of bacteria and cultured following the general protocols on the WormBook [24]. The RNAi Feeding Library was purchased from Open Biosystems Inc. (Huntsville, AL, USA).

### 4.2. DON Treatment and Lifespan Studies

Synchronized eggs were obtained from gravid worms by the bleaching method (The WormBook). L2 worms were harvested by washing them off the plate with sterile water and centrifugation at 300× *g* for 2 min.

L2 worms were treated with DON at different concentrations in 0.6% DMSO in 1 mL S-medium (The WormBook) supplemented with OP50 bacterial cells in 24-well plate at room temperature for 24 h. After DON treatment, worms developed up to the L4 stage and were harvested by centrifugation at 300× *g* for 2 min and washed with water. For each treatment, 30 worms were placed into each well of a 24-well NGM plate supplemented with OP50 bacteria. The number of eggs laid was counted after 24 h. Individual worms were also placed into 24-well plates supplemented with OP50 bacteria and 0.1 mg/mL FUDR (Sigma Aldrich, St. Louis, MO, USA) at 3 worms per well, totally 30 worms per treatment. The viability of worms was recorded by observation under a Nikon SMZ645 stereomicroscope and by assessing the response to mechanical stimulation. All experiments were repeated three times. The student *t*-test was used to assay the significant difference among treatments.

### 4.3. Real-Time Quantitative Polymerase Chain Reaction (RT-qPCR) Analysis of Gene Expression

Worms at the L2 stage were treated with 125, 250 and 500 μg/mL DON for 24 h and were harvested by centrifugation and washed with water. Total RNA was isolated by the freeze/thaw method (The WormBook) using Trizol (Invitrogen, Waltham, MA, USA), and then precipitated with ethanol and resuspended in DEPC (diethyl pyrocarbonate)-treated water. The RNA concentration and quality were measured by a Nanodrop spectrophotometer (Thermo Fisher, Waltham, MA, USA). Reverse transcription (RT) reaction was carried out with the High Capacity cDNA Synthesis Kit (Applied Biosystems/Thermo Fisher, Waltham, MA, USA) using random primers. Real-time quantitative qPCR was conducted with gene-specific primers (Table 1), SYBR Green master mix and the StepOnePlus Real-time PCR System (Applied Biosystems/Thermo Fisher, Waltham, MA, USA). The actin 1 gene (*T04C12.6.2*) was used as the endogenous control gene. Gene expression levels, expressed as fold-changes, were assessed by the 2^−ΔΔCt^ relative quantification method [25].

### 4.4. RNAseq Analysis

For RNAseq analysis, synchronized L1 worms were treated with either 250 μg/mL DON in 0.6% DMSO or 0.6% DMSO alone for 24 h. Total RNA was isolated by the freeze-thaw method as above. Totally, 200 ng of each RNA sample was provided to BGI (Beijing Genomics Institute, Shenzhen, China) for RNAseq analysis. mRNA was enriched on oligo d(T) magnetic beads, and the resulting cDNA library was sequenced via Illumina HiSeq™ 2000. Sequence reads were aligned to the reference genome by Soap 2.21 software. Gene function annotation was performed using BLAST (National Center for Biotechnology Information) and KEGG (Kyoto Encyclopedia of Genes and Genomes), NR (Non-Redundant) and GO (Gene Ontology) databases. Quantification of gene expression was conducted by the RPKM (reads per kilobase per million) algorithm. Pairwise comparison of gene expression levels between DON-treated and DMSO-treated samples was used to identify any differentially expressed genes (DEGs). Relative differences in gene expression difference were expressed as a log2 ratio (representing the fold-change between DON-treated and DMSO-treated samples). P-values were included for each gene. False discovery rate (FDR) ≤0.001 and the absolute value of log2Ratio ≥ 1 were used as the threshold to judge the significance of gene expression difference [26].

### 4.5. Feeding with RNAi Bacteria

To validate the involvement of genes in the response of to DON cytotoxicity, the HT115 (DE3) *E. coli* cells harboring the RNAi (RNA interference) gene knock-down constructs (Open Biosystems, Huntsville, AL, USA) were used to feed *C. elegans*. Synchronized eggs were placed on NGM plates with bacterial lawns of OP50, and *oac-32*, *ugt-26* and *ugt*-RNAi cells for 24 h. L2 worms were washed off the plates with water and then washed with S-medium. Worms were treated with 250 g/mL DON in 1 mL of S-medium supplemented with OP50, and *oac-32*, *ugt-26* or *ugt-28* RNAi bacteria for 24 h. Worms were collected and the number of dead worms was counted in three groups of 50 worms each for each treatment. The experiment was repeated three times. DON-treated and worms fed with different bacteria were then cultured on NGM plate seeded with OP50 lawn to record the lifespan of worms.

## Figures and Tables

**Table 1:** Gene-specific primers used in real-time quantitative polymerase chain reaction (RT-qPCR) analysis (5′- to -3′).

Gene	Forward Primer	Reverse Primer
*C17H12.8*	GGCTCCTTGCACTTGTCACA	ATGATTGGCTCAGGGATCTGA
*K08D8.5*	CCGGGAAGTCGAATGAACAT	GATGCAACACCTGCCAAAGA
*F35E12.5*	CGCAGTTCAGTTGCCCATT	GGGATTGATCCAGACATTCCA
*T04C12.6.2*	CTCCACGCGCCGTGTT	CATACCGACCATGACTCCTTGA

**Table 2:** Genes that were up- (+) or down- (−) regulated by more than 10-fold in response to treatment with 250 μg/mL DON for 24 h.

Gene Symbol	Gene Name by WormBase	Gene Function by WormBase	Fold Change of Gene Expression
*F11D11.3*	*F11D11.3* gene	unknown	612.6 +
*F52F10.4*	*oac-32* (o-acyltransferase homolog)	transferase activity, transferring acyl	513.4 +
*H23L24.5*	*pme-4* [poly(ADP-ribose) metabolism enzyme]	poly(ADP-ribose) glycohydrolase activity	501.6 +
*C07G3.2*	*irg-1* (infection response gene)	defense response to Gram-negative bacterium, innate immune response	94.5 +
*F25A2.1*	*F25A2.1* gene	triglyceride lipase activity	52.0 +
*Y39G10AR.6*	*ugt-31* (UDP-glucuronosyl transferase)	transferase activity, transferring hexosyl groups	39.5 +
*T24A6.7*	*T24A6.7* gene	unknown	37.2 +
*C10H11.6*	*ugt-26* (UDP-glucuronosyl transferase)	transferase activity, transferring hexosyl groups	37.1 +
*Y49C4A.8*	*ugt-29* (UDP-glucuronosyl transferase)	transferase activity, transferring hexosyl groups	33.1 +
*C17H12.8*	*C17H12.8* gene	PMK-1-regulated gene in innate immunity	32.4 +
*T16G1.5*	*T16G1.5* gene	transferase activity, transferring phosphorus-containing groups	28.2 +
*C10H11.4*	*ugt-28* (UDP-glucuronosyl transferase)	transferase activity, transferring hexosyl groups	27.8 +
*F08F3.7*	*cyp-14A5* (cytochrome P450 family)	heme binding, iron ion binding, oxidoreductase activity	27.7 +
*Y58A7A.5*	*Y58A7A.5* gene	unknown	26.7 +
*Y26D4A.21*	*Y26D4A.21* gene	unknown	25.5 +
*C49G7.7*	*C49G7.7* gene	unknown	23.7 +
*Y39A3B.7*	*Y39A3B.7* gene	unknown	23.5 +
*T05F1.9*	*T05F1.9* gene	unknown	23.3 +
*F45E4.1*	*arf-1.1* (ADP-ribosylation factor related)	GTP binding	22.4 +
*F14F9.3*	*F14F9.3* gene	zinc ion binding	21.8 +
*C06B3.7*	C06B3.7 gene	unknown	21.5 +
*F14F9.4*	*F14F9.4* gene	zinc ion binding	20.1 +
*F22E10.1*	*pgp-12* (P-glycoprotein related)	ATP binding, ATPase activity	18.9 +
*ZC239.12*	*sdz-35* (SKN-1 dependent zygotic transcript)	protein binding	17.3 +
*C32H11.1*	*C32H11.1* gene (encoding a CUB-like domain containing protine)	innate immune response to several different bacterial pathogens	17.3 +
*F14F9.2*	*F14F9.2* gene	unknown	16.7 +
*Y119D3A.3*	*fbxa-35* (F-box A protein)	protein binding	16.3 +
*ZK228.4*	*ZK228.4* gene	unknown	15.5 +
*F56D6.2*	*clec-67* (C-type lectin)	carbohydrate binding	14.9 +
*F33H12.7*	*F33H12.7* gene	unknown	14.7 +
*F55G11.5*	*dod-22* (nownstream of DAF-16, regulated by DAF-16)	defense response to Gram-negative bacterium, innate immune response	13.9 +
*F14H8.6*	*cng-1* (cyclic nucleotide gated channel)	ion transmembrane transporter activity	13.7 +
*C49G7.5*	*irg-2* (infection response gene)	defense response to Gram-negative bacterium, innate immune response	13.3 +
*K11D12.5*	*swt-7* (SWEET sugar transporter family)	defense response	13.2 +
*Y58A7A.3*	*Y58A7A.3* gene	zinc ion binding	12.8 +
*F43C1.7*	*F43C1.7* gene	unknown	12.7 +
*C17H12.6*	*C17H12.6* gene	unknown	12.2 +
*F08E10.7*	*scl-24* (SCP-like extracellular protein)	unknown	12.2 +
*F36G9.12*	*oac-20* (o-acyltransferase homolog)	transferase activity, transferring acyl groups other than amino-acyl groups	12.2 +
*Y37H2A.14*	*Y37H2A.14* gene	unknown	12.1 +
*K08D8.5*	*K08D8.5* gene (encoding a CUB-like domain containing protine)	innate immune response to several different bacterial pathogens	12.0 +
*F35E12.5*	*F35E12.5* gene	unknown	11.8 +
*F54D5.4*	*F54D5.4* gene	unknown	10.9 +
*T05B4.8*	*T05B4.8* gene	unknown	10.2 +
W05G11.3	*col-88* (collagen)	structural constituent of cuticle	415.8
T26H2.5	*sqst-3* (SeQueSTosome related)	zinc ion binding	250.4 −
C42D8.2	*vit-2* (vitellogenin structural genes) (yolk protein genes)	lipid transporter activity	44.7 −
C45G7.3	*ilys-3* (invertebrate lysozyme)	lysozyme activity	21.3 −
F41F3.4	*col-139* (collagen)	structural constituent of cuticle	17.7 −
M18.1	*col-129* (collagen)	structural constituent of cuticle	16.4 −
B0218.8	*clec-52* (C-type lectin)	carbohydrate binding	15.4 −
ZK1193.1	*col-19* (collagen)	structural constituent of cuticle	12.9 −
C36A4.1	*cyp-25A1* (cytochrome P450 family)	heme-binding, iron ion binding, oxidoreductase activity	12.8 −
F26F12.1	*col-140* (collagen)	structural constituent of cuticle	12.1 −

## Supplementary Materials

The following are available online at http://www.mdpi.com/2072-6651/10/7/262/s1, Table S1: The complete RNAseq data for all genes of *C. elegans*.
